# Elevated Risk for Antimicrobial Drug–Resistant *Shigella* Infection among Men Who Have Sex with Men, United States, 2011–2015

**DOI:** 10.3201/eid2209.160624

**Published:** 2016-09

**Authors:** Anna Bowen, Julian Grass, Amelia Bicknese, Davina Campbell, Jacqueline Hurd, Robert D. Kirkcaldy

**Affiliations:** Centers for Disease Control and Prevention, Atlanta, Georgia, USA

**Keywords:** Shigella, shigellosis, men who have sex with men, United States, antimicrobial resistance, bacteria, azithromycin, ceftriaxone, fluoroquinolones

## Abstract

*Shigella* spp. cause ≈500,000 illnesses in the United States annually, and resistance to ciprofloxacin, ceftriaxone, and azithromycin is emerging. We investigated associations between transmission route and antimicrobial resistance among US shigellosis clusters reported during 2011–2015. Of 32 clusters, 9 were caused by shigellae resistant to ciprofloxacin (3 clusters), ceftriaxone (2 clusters), or azithromycin (7 clusters); 3 clusters were resistant to >1 of these drugs. We observed resistance to any of these drugs in all 7 clusters among men who have sex with men (MSM) but in only 2 of the other 25 clusters (p<0.001). Azithromycin resistance was more common among MSM-associated clusters than other clusters (86% vs. 4% of clusters; p<0.001). For adults with suspected shigellosis, clinicians should culture feces; obtain sex histories; discuss shigellosis prevention; and choose treatment, when needed, according to antimicrobial drug susceptibility. Public health interviews for enteric illnesses should encompass sex practices; health messaging for MSM must include shigellosis prevention.

## Abstract 2

These men were 3–77 times more likely than other persons to have shigellae resistant to >1 key drugs.

Shigellosis, the third most common human bacterial enteric infection in the United States, causes ≈500,000 illnesses each year (*1*). Because as few as 10 bacteria can cause infection, shigellosis outbreaks typically are large; control can be difficult unless interventions are implemented early in an outbreak (*2*). Although bloodstream infection is uncommon among immunocompetent hosts, shigellosis patients frequently are treated with antimicrobial medications to reduce illness duration and possibly transmission (*3*). High rates of resistance to ampicillin and trimethoprim/sulfamethoxazole have made ciprofloxacin, ceftriaxone, and azithromycin the preferred antimicrobial agents for adults and children with shigellosis; ceftriaxone is also the preferred treatment for invasive shigellosis (*4**–**6*). However, shigellae resistant to these drugs have emerged in the United States and abroad (*7**–**17*). Although shigellosis rates are highest for young children, most reports document ciprofloxacin- or azithromycin-resistant shigellosis largely among men who have sex with men (MSM) (*7**,**8**,**10**,**12**–**17*). Estimates of risk for antimicrobial drug–resistant shigellae among different populations or by transmission route have not been reported. We investigated the associations between transmission route and antimicrobial resistance among US shigellosis clusters reported during 2011–2015.

## Methods

Because sexual transmission data are not captured in the national enteric disease surveillance systems of the Centers for Disease Control and Prevention (CDC), we queried the enteric disease cluster management database of the CDC Outbreak Response and Prevention Branch (ORPB; Division of Foodborne, Waterborne, and Environmental Diseases, National Center for Emerging and Zoonotic Infectious Disease) for all US shigellosis clusters during January 2011–December 2015 (*18**–**21*). This database is not part of a surveillance system; instead, it is used in real time to track and guide response to clusters of enteric infections, including shigellosis, that CDC investigates. It contains unpublished summaries of patient demographics, suspected or confirmed transmission route and vehicles, cluster onset date and duration, and pulsed-field gel electrophoresis (PFGE) patterns associated with the cluster. ORPB identifies shigellosis clusters for inclusion in this database in 3 ways: 1) when CDC’s PulseNet observes a *Shigella* PFGE pattern approximately twice as often as baseline and in >1 US state or territory during a 60-day period, PulseNet assigns a cluster code to that pattern; 2) state health departments can choose to contact CDC about ongoing single-state or multistate clusters; 3) since 2014, CDC also has used data from the National Antimicrobial Resistance Monitoring System for Enteric Bacteria (NARMS), which strives to test every 20th *Shigella* isolate nationally and 3 representative isolates from every shigellosis outbreak, to identify isolates harboring resistance to clinically important antimicrobials (*22**,**23*). CDC then queries PulseNet for other isolates with PFGE patterns indistinguishable from the resistant isolate. For *Shigella*, all identified clusters are added to the ORPB database.

Our analysis comprised clusters with both antimicrobial drug susceptibility data in NARMS and documentation of transmission route or affected population. We linked clusters to NARMS by PulseNet cluster code or local outbreak code to obtain information about the antimicrobial resistance phenotype associated with the cluster. We used standard definitions for ciprofloxacin and ceftriaxone resistance and defined azithromycin resistance as an azithromycin MIC greater than that for wild-type *Shigella* (*24*). Among isolates with phenotypic azithromycin resistance, we used PCR to screen for the macrolide resistance genes *mph*A and *erm*B. We classified a cluster as resistant to >1 of these antimicrobial drugs if any isolate within the cluster harbored resistance to >1 of these drugs.

Few public health jurisdictions routinely collect the MSM status of shigellosis patients, but they might do so through telephone interviews, in-person interviews, medical record reviews, or links to sexual health databases. Public health jurisdictions did not use standardized definitions for MSM-associated transmission; however, we categorized transmission as MSM-associated if any MSM-associated transmission was recorded for a cluster. If subclusters with distinct transmission routes were reported within a single cluster, we analyzed the subclusters as separate clusters. Using Fisher exact test, we compared proportions of clusters with antimicrobial resistance by transmission category.

## Results

Of 49 clusters reported during 2011–2015, we excluded 19 (39%) because of insufficient data (transmission data but no antimicrobial resistance data, 5 clusters; neither transmission data nor antimicrobial resistance data, 14 clusters). Of the 30 (61%) remaining clusters, 2 encompassing subclusters with MSM-associated and other person-to-person transmission were further divided by transmission category, for a total of 32 clusters eligible for analysis (Table 1). Suspected or confirmed transmission was reported to involve child care centers, camps, or schools (10 clusters); MSM (7 clusters); other person-to-person transmission (7 clusters); food (6 clusters); or recreational water (2 clusters). Nine clusters were caused by shigellae resistant to at least 1 of the following: ciprofloxacin (3 clusters), ceftriaxone (2 clusters), or azithromycin (7 clusters). Three of these clusters were resistant to >1 of these drugs. At least 1 isolate from each azithromycin-resistant cluster was confirmed to harbor the macrolide resistance genes *mph*A or *erm*B. Of the 9 antimicrobial drug–resistant clusters, 8 were caused by *S. sonnei* and 1 by *S. flexneri*. Isolates from all 7 MSM-associated clusters harbored resistance to >1 of these drugs (Table 1). The prevalence of resistance among MSM-associated and other clusters significantly differed for ceftriaxone (p = 0.04), azithromycin (p<0.001), azithromycin and either ciprofloxacin or ceftriaxone (p = 0.007), and any of these 3 drugs (p<0.001) (Table 2). The proportion of MSM-associated clusters with resistance to any of these 3 drugs (i.e., ciprofloxacin, ceftriaxone, or azithromycin) was 3–77 times greater, and the proportion with resistance to azithromycin was 3–500 times greater, than the proportion of other clusters with such resistance phenotypes. None of 10 clusters associated with child care, camps, or schools was caused by ciprofloxacin-, ceftriaxone-, or azithromycin-resistant strains.

**Table 1:** Clusters of shigellosis with resistance to CIP, CRO, or AZM, United States, January 2011–December 2015*

Cluster	PFGE pattern†	*Shigella* sp.	Onset	No. cases‡	No. states with cases	Transmission route	Median patient age, y	Male sex, %	No. isolates tested by NARMS	No. isolates with resistance§																No. drug classes with resistance§	Ref
										Penicillins, AMP	β-lactam inhibitors, AMC	Cephems			TETs, TET	Aminoglycosides			Quinolones			Folate pathway inhibitors		Phenicols, CHL	Macrolides, AZM		
												FOX	CEF	CRO		STR	GEN		NAL	CIP		SXZ	TMP/SXT				
With resistance to AZM, CRO, or CIP																											
1	J16X01.3056, J16X01.7056	*S. sonnei*	2012 Apr	43	1	Food (bridge club)	87	57	4	0	0	0	0	0	4	4	0		0	0		4	4	0	4	4	(*11*)
2	J16X01.1244, J16X01.0728, J16X01.0038	*S. sonnei*	2014 Apr	128	16	MSM	38	83	14	11	0	3	3	3	11	14	0		0	0		12	12	0	10	6	
3	J16X01.0618	*S. sonnei*	2014 Apr	7	6	MSM	36	100	4	3	0	0	0	0	4	4	0		0	0		4	4	0	3	5	
4	J16X01.0232, J16X01.0775	*S. sonnei*	2014 May	>300	34	Person-to-person	43	54	32	1	0	0	0	0	26	26	0		32	13		26	26	0	0	5	(*9*)
5	J16X01.3176, J16X01.3056	*S. sonnei*	2014 Jul	24	2	MSM	36	100	21	21	0	0	0	0	21	21	0		0	0		21	21	0	21	5	(*8*)
6	J16X01.0232, J16X01.0775	*S. sonnei*	2014 Sep	22	5	MSM	42	90	12	11	0	0	0	0	12	12	0		12	12		12	12	0	11	6	(*9,* *10*)
7	J16X01.0286, J16X01.3294	*S. sonnei*	2015 Mar	12	7	MSM	37	100	2	0	0	0	0	0	1	1	0		2	2		1	1	0	0	4	
8	J16X01.1058, J16A26.0030	*S. sonnei*	2015 Apr	41	9	MSM	26	85	7	6	0	0	6	5¶	1	7	0		0	0		7	7	0	6	6	
9	JZXN11.0302	*S. flexneri* 4a (IV:-)	2015 Aug	6	3	MSM	40	100	3	3	0	0	0	0	3	3	0		0	0		3	3	3	1	6	
Without resistance to AZM, CRO, or CIP																											
10	J16X01.0408, J16A26.0071	*S. sonnei*	2011 Jul	16	1	Recreational water	9	44	1	0	0	0	0	0	1	1	0		0	0		1	1	0	0	3	
11	J16X01.1290	*S. sonnei*	2011 Sep	61	1	Person-to-person	7	41	5	0	0	0	0	0	0	5	0		0	0		0	0	0	0	1	
12	J16X01.0283, J16A26.0051	*S. sonnei*	2011 Nov	12	2	Food	49	92	1	0	0	0	0	0	0	1	0		0	0		0	1	0	0	2	
13	J16X01.0559	*S. sonnei*	2012 Feb	33	1	Person-to-person	NA	NA	3	3	0	0	0	0	0	3	0		0	0		3	3	0	0	3	
14	J16X01.0283, J16X01.0199	*S. sonnei*	2012 Mar	18	1	Person-to-person	9	25	1	1	0	0	0	0	0	1	0		0	0		0	0	0	0	2	
15	J16X01.1986, J16X01.0199, J16X01.2995	*S. sonnei*	2012 Jul	100	1	Person-to-person	3	NA	3	0	0	0	0	0	0	3	0		0	0		0	0	0	0	1	
16	J16X01.3073, J16X01.3072	*S. sonnei*	2012 Oct	29	1	Person-to-person	8	46	4	0	0	0	0	0	0	4	0		0	0		0	0	0	0	1	
17	J16X01.2995	*S. sonnei*	2013 Mar	13	1	Person-to-person	4	33	5	1	0	0	0	0	0	5	0		0	0		1	1	0	0	3	
18	J16X01.0408, J16X01.0981, J16X01.3218	*S. sonnei*	2013 Jul	58	4	Recreational water	8	48	6	0	0	0	0	0	5	6	0		0	0		5	5	0	0	3	
19	J16X01.3157	*S. sonnei*	2014 Feb	263	3	Person-to-person	7	48	19	2	1	0	0	0	3	19	0		0	0		4	7	0	0	4	
20	J16X01.1166	*S. sonnei*	2014 Mar	9	1	Food	31	56	2	0	0	0	0	0	2	2	0		0	0		2	2	0	0	3	
21	J16X01.2624	*S. sonnei*	2014 Jul	8	3	Food	25	34	2	0	0	0	0	0	2	2	0		0	0		2	2	0	0	3	
22	J16X01.0199, J16X01.0283	*S. sonnei*	2014 Jul	335	26	Person-to-person	8	45	21	1	0	0	0	0	6	21	0		0	0		7	14	0	0	3	
23	J16X01.1166, J16X01.1148	*S. sonnei*	2014 Jul	474	31	Person-to-person	8	42	59	7	0	0	0	0	2	59	0		1	0		3	4	0	0	3	
24	J16X01.2493, J16X01.0623	*S. sonnei*	2014 Sep	30	5	Food	28	31	5	0	0	0	0	0	3	5	0		0	0		3	4	0	0	3	
25	J16X01.1254	*S. sonnei*	2014 Oct	13	5	Person-to-person	17	45	4	3	0	0	0	0	0	4	0		0	0		0	0	0	0	2	
26	J16X01.0437, J16X01.0466	*S. sonnei*	2014 Nov	53	4	Person-to-person	9	51	5	0	0	0	0	0	1	3	0		0	0		1	3	0	0	3	
27	J16X01.1244	*S. sonnei*	2014 Jun	6	1	Food	13	50	1	0	0	0	0	0	0	1	0		0	0		1	1	0	0	2	
28	J16X01.1166, J16X01.1148, J16X01.2427	*S. sonnei*	2015 May	982	26	Person-to-person	7	44	42	16	0	0	0	0	3	42	0		0	0		3	5	0	0	3	
29	J16X01.1278	*S. sonnei*	2015 Jun	34	3	Person-to-person	7	53	4	0	0	0	0	0	0	4	0		0	0		0	0	0	0	1	
30	J16X01.2261	*S. sonnei*	2015 Jun	15	3	Person-to-person	4	38	3	0	0	0	0	0	0	3	0		0	0		0	0	0	0	1	
31	J16X01.1298	*S. sonnei*	2015 Jul	15	4	Person-to-person	21	30	1	0	0	0	0	0	0	1	0		0	0		0	1	0	0	2	
32	≈27 patterns	*S. sonnei*	2015 Oct	118	1	Person-to-person	26	50	7	0	0	0	0	0	3	7	0		0	0		3	4	0	0	3	

*AMC, amoxicillin/clavulanic acid; AMP, ampicillin; AZM, azithromycin; CEF, ceftiofur; CHL, chloramphenicol; CIP, ciprofloxacin; CRO, ceftriaxone; FOX, cefoxitin; GEN, gentamycin; MSM, men who have sex with men; NAL, nalidixic acid; NARMS, National Antimicrobial Resistance Monitoring System for Enteric Bacteria; NA, not available; PFGE, pulsed-field gel electrophoresis; Ref, reference; STR, streptomycin; SXZ, sulfisoxazole; TET, tetracycline; TMP/SXT, trimethoprim/sulfamethoxazole. Blank cells indicate no previous reference, i.e., clusters that have not previously been published. †PFGE was performed by PulseNet using enzyme XbaI for *S. sonnei* and NotI for *S. flexneri* (*22*). ‡Clusters 2, 6, and 8 had ongoing transmission as of December 2015. §As defined by the Clinical and Laboratory Standards Institute; for AZM, resistance was defined as non–wild-type MICs (*24*). ¶Among 6 isolates tested.

**Table 2:** Differences in antimicrobial resistance phenotype by transmission route among clusters of *Shigella* infection, United States, January 2011–December 2015

Antimicrobial resistance phenotype	MSM-associated transmission, no. (%, 95% CI†), n = 7	Transmission other than MSM-associated, no. (%, 95% CI†), n = 25	p value‡
CIP	2 (29, 5–67)	1 (4, 0.2–18)	0.1
CRO	2 (29, 5–67)	0 (0, 0–11)	0.04
AZM	6 (86, 47–99)	1 (4, 0.2–18)	<0.001
AZM, CIP, or CRO	7 (100, 65–100)	2 (8, 1.3–24)	<0.001
AZM and either CIP or CRO	3 (43, 12–78)	0 (0, 0–11)	0.007

*AZM, azithromycin; CIP, ciprofloxacin; CRO, ceftriaxone; MSM, men who have sex with men. †Mid-p exact 95% CI of the percentage resistant. ‡By 2-tailed Fisher exact test.

## Discussion

All shigellosis clusters that we identified among MSM in the United States during 2011–2015 were caused by strains resistant to >1 of the preferred antimicrobial agents for shigellosis. Although our sample was small, the estimated prevalence of resistance to preferred antimicrobial drugs for MSM-associated shigellosis clusters was 3–77 times the prevalence for clusters with nonsexual transmission routes. Although shigellosis with these antimicrobial drug resistance phenotypes has been documented among MSM internationally, the reasons for this association are unknown (*7**,**8**,**12**–**14**,**16**,**17*). Additional studies are needed to elucidate these findings; identify specific risk factors; understand clinical outcomes for patients infected with these resistant strains and in the setting of HIV infection; and develop effective interventions to prevent infection of MSM with shigellae, particularly drug-resistant shigellae.

Although our results suggest that shigellae with resistance to ciprofloxacin, ceftriaxone, or azithromycin circulate predominantly among MSM in the United States, these strains are likely to emerge among other populations. Efforts to facilitate improved hygiene practices among persons at high risk for shigellosis or at high risk for transmitting shigellosis to others (e.g., child care attendees, staff, and parents; marginally housed persons; international travelers; and food handlers) are needed now to limit transmission when multidrug-resistant *Shigella* strains inevitably begin circulating among these populations.

Although the associations we found between antimicrobial drug–resistant shigellosis and transmission route (i.e., MSM-associated transmission vs. other transmission) are striking, this analysis has several limitations. Because the ORPB cluster management database is used to guide response rather than as a formal reporting system, it is likely to contain only a small fraction of shigellosis clusters, and the details about each cluster are not always complete. Furthermore, many public health jurisdictions do not routinely perform PFGE on shigellae, making it difficult to detect clusters and, when a cluster is detected, to locate all cases associated with the cluster. Therefore, clusters included in this analysis might not be representative of all US shigellosis clusters, and the number of cases reported per cluster is likely to be smaller than reality. Additionally, nearly 40% of clusters were not analyzed because of missing data. However, the selection of clusters in this analysis is unlikely to have biased the association between antimicrobial drug resistance phenotype and transmission route. Next, both antimicrobial drug resistance phenotypes and transmission routes are likely to be heterogeneous within each cluster. We also lacked information about patients’ antecedent exposure to antimicrobial drugs, HIV infection, and other factors that might enhance our findings, and we did not have access to clinical treatment or outcome data. Finally, because of the small sample size, we cannot reliably compare the prevalence of resistance among MSM-associated clusters and other clusters. Nonetheless, the markedly higher prevalence of such resistance observed in our study is concerning and warrants future study.

The increasing use of PCR-based, culture-independent diagnostic tests for *Shigella* will make it difficult to identify cases and clusters of multidrug-resistant shigellosis and to provide laboratory-informed antimicrobial treatment (*25*). When shigellosis is suspected or when a culture-independent diagnostic test suggests *Shigella* infection, clinicians should culture fecal specimens and test *Shigella* isolates for antimicrobial drug susceptibility, including susceptibility to azithromycin (*24*). Patients who do not improve with treatment should be re-cultured. Further characterizing isolates by PFGE or whole-genome sequencing will assist health departments with cluster detection and control.

Alternative treatment options are limited for persons infected with ciprofloxacin-, ceftriaxone-, or azithromycin-resistant shigellae, making shigellosis prevention critical. Clinicians should counsel all patients with suspected shigellosis about meticulous handwashing and hygiene (*26*). Among adult patients, sexual histories focused on sex of recent partners and specific sex practices can inform empiric treatment and indicate a need for further testing and counseling to prevent sexual transmission of shigellosis (*27*). If treatment is required, drug choice should be informed by the results of antimicrobial susceptibility testing. During public health interviews for enteric illnesses, routinely collecting sex of sex partners and, among those with multiple recent partners, where patients find partners would improve understanding of risk groups and permit more targeted interventions. Surveillance and interventions for shigellosis among MSM would be strengthened by more intensive collaboration between enteric disease and sexually transmitted disease programs within public health departments. Health messaging for MSM should include information about risk for and prevention of shigellosis and drug-resistant shigellosis (*26**,**27*).
